# Using national movement databases to help inform responses to swine disease outbreaks in Scotland: the impact of uncertainty around incursion time

**DOI:** 10.1038/srep20258

**Published:** 2016-02-01

**Authors:** Thibaud Porphyre, Lisa A. Boden, Carla Correia-Gomes, Harriet K. Auty, George J. Gunn, Mark E. J. Woolhouse

**Affiliations:** 1 Centre for Immunity, Infection and Evolution, University of Edinburgh, King’s Buildings, Edinburgh, UK; 2 School of Veterinary Medicine, Boyd Orr Centre for Population and Ecosystem Health, College of Medical, Veterinary and Life Sciences, University of Glasgow, Glasgow, UK; 3 Epidemiology Research Unit, SRUC, Drummondhill, Stratherrick Road, Inverness, UK

## Abstract

Modelling is an important component of contingency planning and control of disease outbreaks. Dynamic network models are considered more useful than static models because they capture important dynamic patterns of farm behaviour as evidenced through animal movements. This study evaluates the usefulness of a dynamic network model of swine fever to predict pre-detection spread via movements of pigs, when there may be considerable uncertainty surrounding the time of incursion of infection. It explores the utility and limitations of animal movement data to inform such models and as such, provides some insight into the impact of improving traceability through real-time animal movement reporting and the use of electronic animal movement databases. The study concludes that the type of premises and uncertainty of the time of disease incursion will affect model accuracy and highlights the need for improvements in these areas.

The epidemics of bovine spongiform encephalopathy in Europe^1^ and of foot-and-mouth disease in the UK^2^ showed the importance of using mathematical models of disease transmission in providing key information to design contingency planning for animal disease outbreaks. By providing epidemiological insight that can be considered alongside the complex interactions between social, economic and welfare outcomes of disease incursions and control strategies, models have helped to inform decisions on disease control^2^,^3^,^4^,^5^, and can also be used judiciously as tools to improve communication with non-expert stakeholders^6^. Models must be based on robust data and assumptions to usefully inform policies and add value to field-based control activities. However, disease control decisions during epidemic responses are made in the context of wide range of uncertainties. Improving our understanding of the impact of these uncertainties on infectious disease models outcomes is therefore a way to improve their capabilities to efficiently inform policy.

Network models, which were once confined to physics and social science problems^7^, have proliferated in the field of human^8^,^9^,^10^ and animal^4^,^5^,^11^,^12^ health and are increasingly used to inform disease control strategies as part of national contingency plans. When applied to animal diseases, these models consider farms as nodes of a network that are linked by the transfer or movement of (potentially infected) animals. Animal movements are increasingly recorded in national databases, informing on the daily number of animals moved between all farms present in an industry. This large volume of data enables models to appropriately capture the dynamic changes in the contact structure between farms, and therefore enables them to directly adjust for the underlying farm-level economic and behavioural variations when moving animals. As such, predictions from dynamic networks models are potentially more accurate than those from models considering the animal movement network as static^13^,^14^.

As movement of animals within the livestock industry carries the risk of transmitting infectious diseases across substantial geographical distances, dynamic network models have been increasingly used prior to disease outbreaks to improve preparedness. Particularly, dynamic network models have been used to assess the potential for pre-detection spread of infection via movements of animals^5^,^11^, identify regional and local movement patterns^4^,^11^, and provide guidance for the design of efficient control and surveillance strategies^4^,^12^. However, their use may go further, notably by estimating the extent of the disease spread that has already occurred when disease incursions have been detected and restrictions on animal movements are implemented^15^. By quickly and accurately estimating the spatial extent of the pre-detection spread via movements of animals, they potentially offer additional tools to support field-based contact tracing, and increase the efficiency of disease control responses. However, little work has been done to exploit dynamic network models to such effect.

The emphasis on using dynamic network models for contingency planning, but not during an outbreak, may be due to an assumption that they are less useful for making predictions of disease spread or identifying high risk farms in scenarios in which disease incursion has already occurred^6^,^16^. This assumption may be based on two prior beliefs: (*i*) that data quality may be compromised by time-lags in data recording; and (*ii*) that the date of infection, which is critical to appropriate data selection, may be difficult to ascertain with any certainty. Time-lags in data recording would mean that models have to rely on historical data. However, this problem has been minimised by the advent of electronic databases which mean farmers may directly report movements ahead of time. As a result, live animal movements, such as for sheep and pigs, are now available in real-time in Scotland (through the Scottish livestock electronic identification and traceability database ScotEID, https://www.scoteid.com/) to inform epidemiological modelling to predict the dissemination of a pathogen throughout the livestock industry in a timeframe relevant to disease control activities.

Establishing an accurate date of infection is crucial for identifying which data should be included in the model. This can be difficult, as it depends on factors such as clinical presentation and the success of field-based contact tracing procedures, both of which can vary widely. The impact of this uncertainty around date of infection may depend on the temporal dynamism in the pattern of animal movements between farms, and differences in farm trading behaviour, in a given livestock industry. This may affect model predictions (and the uncertainty around them) of the patterns of disease spread.

The objective of this study is to assess the usefulness of dynamic network models for predicting the spatial extent of the pre-detection spread via movements of animals, when there may be considerable uncertainty surrounding the time of incursion of infection. In order to achieve this objective, we have focused on diseases of pigs (e.g. swine fevers such as classical swine fever (CSF) or African swine fever (ASF) viruses) which have non-specific clinical signs as well as a high potential to be transmitted through animal movements^17^,^18^. These characteristics provide a useful model scenario because of the challenging nature of disease detection and the increased potential for silent spread within the pig population. We then explored the usefulness and limitations of using pig movement data (using ScotEID as an exemplar) to inform models when attempting to respond to an infectious disease incursion. Thus the results of this study should also provide insight into the impact of improving traceability through real-time animal movement reporting and the use of electronic animal movement databases.

## Results

### Impact of uncertainty in infection time

We looked at the extent to which inaccuracy in defining the disease incursion date may impact on the accuracy of predictions of pre-detection spread of acute swine diseases via movements of pigs. A premises-based model was developed to simulate their spread through the Scottish swine industry via movements of pigs, in which gathering places (such as markets, and collection centres) were explicitly modelled together with pig producers. In the first instance, we considered the extreme case where infection occurs if at least 1 animal from an infected premises is received by a susceptible one. In this situation, the “infection paths” *Γ*_*t,i*_ of farms that were infected via movements of animals from a single pig producer *i* was computed for each Monday of the year 2012. Here, we considered all *i*^th^ producers that were active during the period [*t*_0_*, t*_0_+*T*] eligible to be an index-case, where *t*_0_ is the incursion date and *T* is the “pre-detection period” (that is, the period between the date of the incursion *t*_0_ and the date of the first detection of the disease). We then compared the infection paths *Γ*_*t,i*_ with those *Γ*_*t*+*δ,i*_ generated when time of infection *t*_0_ is inaccurately estimated by an error *δ* ranging from −7*δ*_0_ to 7*δ*_0_. In this study, infection path *Γ*_*t,i*_ refers to the “correct” full epidemic tree that is generated by a single infection event at time *t*_0_ and left freely spreading for the pre-detection period [*t*_0_*, t*_0_+*T*], while *Γ*_*t*+*δ,i*_ refers to the “predicted” full epidemic tree when the incursion date is inaccurately estimated and for which the pre-detection period is [*t*_0_+*δ, t*_0_+*T–δ*]. We considered, *δ*_0_ = 7 days and *T* = 60 days^19^.

**Figure 1:**
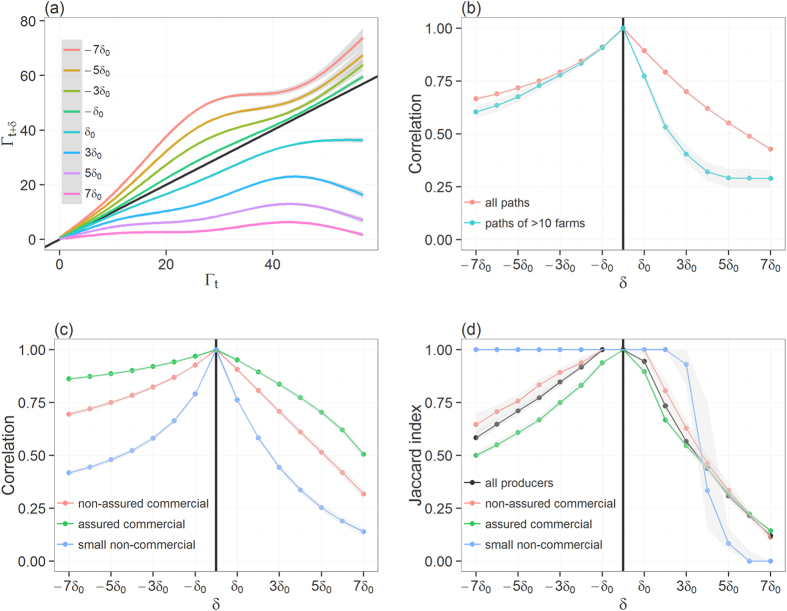
Comparison between the correct infection path and predicted paths generated when the error *δ* in the time of the incursion ranges from −7*δ*_0_ to 7*δ*_0_. (**a**) Lines plot showing the smoothed size of the predicted full epidemic tree *Γ*_*t*+*δ,i*_ as a function of the size of the correct full epidemic tree *Γ*_*t,i*_. (**b**) Changes in the Spearman correlation coefficient between the size of *Γ*_*t,i*_ and that of *Γ*_*t*+*δ,i*_ as a function of the error *δ* in the time of the incursion. Correlation coefficients are computed either upon all generated infection paths or upon infection paths of >10 infected premises. (**c**) Changes in the Spearman correlation coefficient between the size of *Γ*_*t,i*_ and that of *Γ*_*t+δ,i*_ as a function of *δ* and stratified by the producer type of the index-case. (**d**) Quality of infection path prediction, as measured by the median Jaccard similarity index, as a function of *δ* and stratified by the producer type of the index-case. Shaded areas around each line shown in (**a–d**) represent their respective confidence interval. Here, *δ*_0_ = 7days. Diagonal solid line in (**a**) indicates perfect concordance between the true and predicted length of infection paths. The vertical solid line in (**b–d**) indicates the time of the correct incursion time.

In Fig. 1, we show how increasing uncertainty around the time of incursion may affect one’s ability to accurately predict not only the number of premises involved in the full epidemic tree but also their identity. Overall, progressively increasing the error *δ* around the time of the incursion from *δ*_0_ to 7*δ*_0_ yielded a marked reduction in the correlation between sizes (i.e., the number of premises involved in) of infection paths *Γ*_*t,i*_ and *Γ*_*t*+*δ,i*_ (Fig. 1a,b). Although this reduction was consistent across paths of all sizes (Fig. 1a), it was more pronounced for paths of larger sizes (Fig. 1b). Also, there was a clear divide between infection paths generated from commercial producers and those generated from non-commercial producers (Fig. 1c). Despite a wide uncertainty on the time of the incursion, the correlation remained high between infection paths generated by commercial producers (Spearman’s correlation coefficient *ρ* > 0.60), whether assured or non-assured, for errors ranging from −7*δ*_0_ to 4*δ*_0_. In contrast, correlation between infection paths becomes weaker for incursions in non-commercial producers, with *ρ* < 0.60 for errors of ±3*δ*_0_.

In order to see if we could accurately predict which individual premises would be involved in epidemics despite some inaccuracy in the incursion time, we compared the concordance between infection paths *Γ*_*t,i*_ and *Γ*_*t*+*δ,i*_ generated from the same index-case *i*, by calculating the Jaccard similarity index *J*(*Γ*_*t,i*_,*Γ*_*t*+*δ,i*_). The Jaccard index measures the fraction of common premises within paths |*ξ*_*t,i*_ ∪ *ξ*_*t*+*δ,i*_| among the total number of premises |*ξ*_*t,i*_ ∩ *ξ*_*t*+*δ,i*_| involved in both paths. Here, we only focused on infection paths involving more than 10 infected premises.

Progressively increasing the error around the infection time up to 7*δ*_0_ revealed a reduction in the median degree of overlap between paths (Fig. 1d). The rate of this reduction differed, however, whether the incursion time is believed earlier (i.e. *δ* < 0) or later (i.e. *δ* > 0) than the true one. Overall, an error of −4*δ*_0_ in the infection time yielded 77% (95% CI 0.76–0.79) overlaps between the true and predicted paths, whereas an error of >2*δ*_0_ is enough to create completely different paths with paths involving, on average, less than half of common premises.

**Figure 2:**
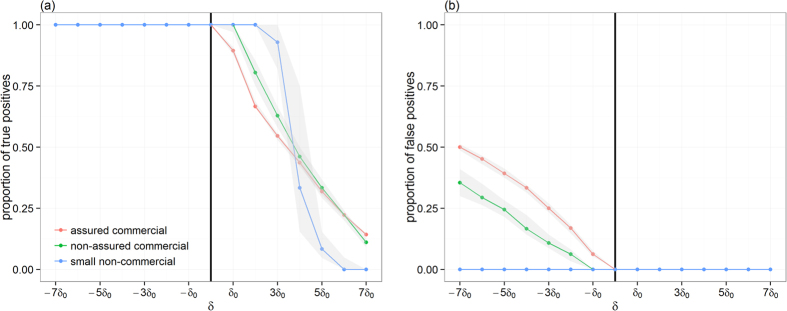
Proportions of true (a) and false (**b**) positives between the correct full infection path and predicted paths generated when the error *δ* in the time of the incursion ranges from −7*δ*_0_ to 7*δ*_0_. Here, *δ*_0_ = 7days. Points/lines represent the observed median proportions, stratified as a function of the producer type of the index-case, whereas shaded areas represent their respective 95% confidence interval. Only infection paths of >10 infected premises are used. The vertical solid line indicates the time of the correct incursion time. The proportion of true positives measures the fraction of common premises within paths |*ξ*_*t,i*_ ∪ *ξ*_*t+δ,i*_| among the number of premises |*ξ*_*t,i*_| that are on the correct path. The proportion of false positives measures the fraction of uncommon premises within paths 1 −|*ξ*_*t,i*_ ∪ *ξ*_*t+δ,i*_| among the number of premises |*ξ*_*t*+*δ,i*_| that are on the wrong path.

Unsurprisingly, variations between producer types were observed in the degree of overlap between *Γ*_*t,i*_ and *Γ*_*t*+*δ,i*_. While the degree of overlap between predicted and the true paths generated by commercial producers followed closely the general trend, it differs greatly when considering paths generated by non-commercial small producers. This was expected, because most paths of >10 infected premises have been generated by commercial producers. However, differences between the degree of overlap for paths generated by commercial and those by non-commercial producers depends on the direction of the error *δ*: when *δ* < 0, predicted paths generated by non-commercial producers have a greater number of common premises with the true path, whereas paths would show a completely different pattern (i.e. *J*(*Γ*_*t,i*_,*Γ*_*t*+*δ,i*_) < 10%) from >4*δ*_0_ (Fig. 1d). These results suggest that if incursion occurs in non-commercial producers, conservative estimates in incursion times would be preferential. However, this may not be true for incursion occurring in commercial producers as a trade-off may exist between optimising the proportion of premises that are truly on the infection path (true positives) and minimising the proportion of premises that are not (false positives). Figure 2 explores how these two epidemiological measures vary with *δ* for paths generated by the different producer types. Over-estimating incursion times for outbreaks generated from commercial producers (whether assured or not), would increase the risk of misclassification. For example, inferences generated for outbreaks from non-assured and from assured commercial producers when *δ*  = *−5δ*_0_ would involve 24% (95% CI 22–29%) and 39% (95% CI 37–41%) of false positives, respectively (Fig. 2b).

**Figure 3:**
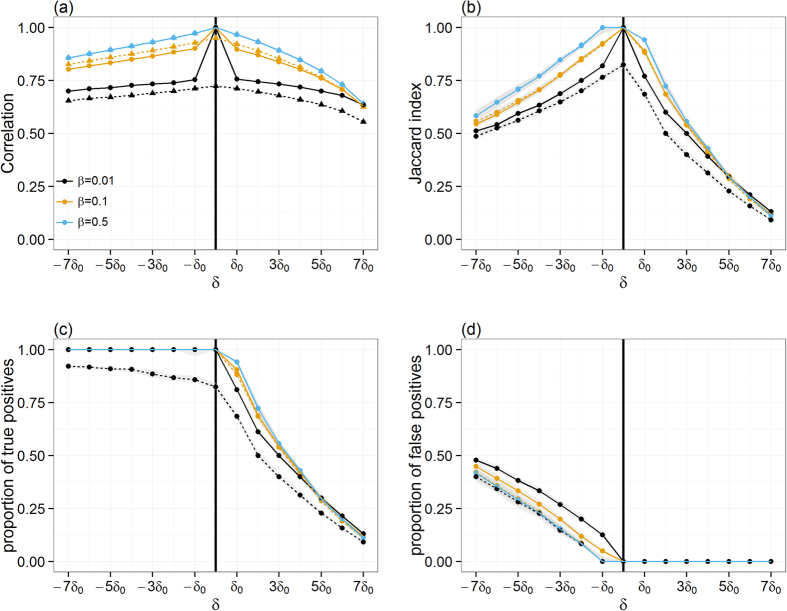
Comparison between the correct infection path and paths generated when the error *δ* in the time of the incursion ranges from −7*δ*_0_ to 7*δ*_0_ and when imperfect transmission occurs. (**a**) Changes in the Spearman correlation coefficient between the size of the correct infection path and paths generated when both the error *δ* in the time of the incursion and the transmission probability *β* vary. Quality of infection path prediction, as measured by the median Jaccard similarity index (**b**), proportion of true **(c**) positives and proportion of false positives (**d**) between the correct infection path and paths generated when both *δ* and *β* vary. Solid and dotted lines indicate how measures may change when comparing predicted partial infection path *Γ’*_*t*+*δ,i*_ to either the correct partial epidemic tree *Γ’*_*t,i*_ generated with *δ* = 0 and *β* < 1 (solid) or the correct full epidemic tree *Γ*_*t,i*_ generated with *δ* = 0 and *β* = 1 (dotted). Shaded areas around each line shown in (**b–d**) represent the confidence interval around the median. Here, *δ*_0_ = 7days. The vertical solid line in (**a–d**) indicates the time of the correct incursion time.

So far in this analysis, the potential for spread of infection via movements of animals has been evaluated considering that any movement from infected premises during the pre-detection period would result in disease transmission to susceptible farms. In reality, the prevalence of disease within infected premises will determine what proportion *β* of its livestock becomes infectious. This, together with the number of animals that are being moved off, will determine what proportion of movements will contain infectious animals. To gain general insight and ensure robustness of the results to variation in *β*, 10,000 simulations for each Monday of the year 2012 with a random index-case per simulation were carried (i.e., total of 570,000 simulations). For each incursion date *t*_0_, the infection paths *Γ’*_*t,i*_ of farms that were infected via animal movements from a single pig producer *i* was then computed and compared to the infection paths *Γ’*_*t*+*δ,i*_ that were predicted when an error *δ* around the time of the incursion is made. As above, *Γ’*_*t,i*_ and *Γ’*_*t*+*δ,i*_ are the “correct” and “predicted” partial epidemic tree, respectively, and correspond to all farms that have a non-null probability of being infected via animal movements from a single pig producer *i*. Figure 3 shows that, whether comparison is made with the “correct” full epidemic tree *Γ*_*t,i*_ (i.e. when *β* = 1) or with the “correct” partial epidemic tree *Γ’*_*t,i*_ (i.e. when *β* < 1), qualitatively similar results as in Fig. 1 are obtained. However, it further appears that decreasing the value of *β* would reduce the effect of *δ* when predicting the size of the infection path (Fig. 3a). It is to note, however, that this result may give a false sense of security as the degree of overlaps between correct and predicted paths still sharply decreases with increasing error *δ* around the time of the incursion from *δ*_0_ to 7*δ*_0_ (Fig. 3b).

### Intrinsic structure of infection paths

**Figure 4:**
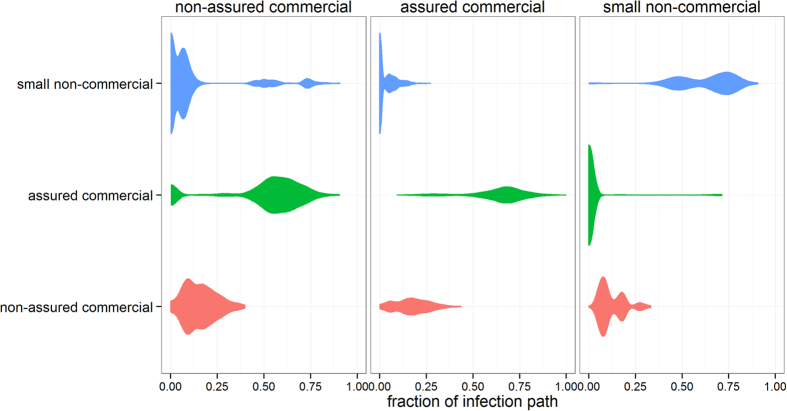
Proportion of non-assured commercial, assured commercial and small non-commercial producers involved in infection paths of >10 infected premises generated by each producer type. Here, columns indicate the producer type of the index-case, whereas rows indicate the type of the producers that are involved in each infection path. The thickness of the shapes is proportional to the density of data points along the x-axis.

Although our findings suggest that inferring the spread of an epidemic from dynamic network models is precarious when the date of the disease incursion is unknown, infection paths may have some intrinsic structure which may still guide contact tracing procedures. Previously, such a structure was found in the Italian cattle industry by comparing epidemic trees and regrouping index-cases which generated similar trees, thereby providing critical information to optimize surveillance systems and define rapid containment strategies^4^. Applying a similar method for the Scottish swine industry, however, would only result in regrouping producers that belong to the same business or are part of the same breeding pyramid. Instead, we looked at the producer type of both the index case and all farms that have been infected via the movement of animals when considering *β* = 1, and determined, for all full epidemic tree *Γ*_*t,i*_ that gave rise to at least 10 cases from the year 2012, the proportion of producers of each type that were involved in each infection path. The results are summarized in Fig. 4.

If disease incursion occurs in the herd of a small producer, the mean risk of disease spillover into assured producers is low (0.011); and similar to the mean risk of disease spillover from assured producers to small producers (0.032). Epidemics which start in a small producer spread into at least one assured producer in only 1.9% of the incursions. However, once an assured producer is infected, 60% (Q1–Q3: 17–71%) of the premises in the generated infection paths would belong to assured producers. In contrast, epidemics generated from assured producers would spread into small producers in 39% of the incursions, but would not involve many of them, with only 8% (Q1–Q3: 2–27%) of premises in these infection paths belonging to small producers. These findings are the consequence of producers adhering to quality assurance scheme guidelines on risks associated with animal trading^20^, confirming that excluding interactions with producers that have lower biosecurity standards is a good biosecurity practice^21^. Such a result may constitute a basis for the development of qualitative rules modulating surveillance activities in the face of an epidemic.

Non-assured commercial producers appear to have a totally different epidemiological profile (Fig. 4). Non-assured commercial producers have a consistently high probability (>95%) of being on an infection path and make up, on average, 17% (Q1–Q3: 9–22%) of premise in these paths, regardless of the producer type of the incursion. In addition, epidemics generated by non-assured producers show a high likelihood of infecting both small producers (0.62) and assured producers (0.85). This result highlights that Scottish swine producers who are commercially driven but do not belong to assurance schemes may potentially represent “epidemiological” bridges between non-commercial and commercial partners, likely because they implement lower biosecurity, particularly with regard to sourcing and sending pigs, compared to assured commercial producers. Therefore, improving biosecurity and targeting surveillance to non-assured producers may be particularly beneficial to optimise responses to disease incursions.

## Discussion

In order to improve preparedness for disease incursion, it is critical to have some understanding of model resiliency to uncertainties which fundamentally underlie the stochastic nature of disease control activities. In this study, we evaluated the resilience of dynamic network models in predicting disease spread after disease incursion, when there may be considerable uncertainty surrounding the timing of infection. A model which predicts the spread of swine fevers was chosen as an exemplar because of the characteristics of the disease and its parameterisation using pig movement data from an electronic database. This has particular relevance and potential policy impact because ASF virus has recently spread within the eastern European region^22^ and the middle east^23^, and now poses an imminent threat to the European swine industry^24^,^25^. Although there are measures in place to reduce the risk of introduction of disease, such as restrictions on the movement of live pigs and animal products in affected areas, and regulations on animal swill feeding (which has been banned in the European Union since 2002), further incursions and spread of these diseases throughout Europe are considered likely^24^,^26^,^27^.

Our analysis not only confirmed that increasing the uncertainty around the incursion date significantly reduced the ability of dynamic network models to predict epidemic characteristics, such as epidemic size, or specific premises that become infected, but also quantified the magnitude of the loss of accuracy of predictions. For example, erroneously estimating the time of incursion more than three weeks earlier appears to generate a low accuracy of predicting cases (i.e. less than 60%, Fig. 1d), which would miss between 30% to 50% of the potentially infected farms (Fig. 2a). Although such a measurement bias may potentially generate longer and more severe epidemics, it may be preferable to the alternative misclassification error. A prediction that a farm is potentially infected, when it is not likely to be because of the true absence of contact with an at-risk farm, may have unintended negative consequences for resource allocation (of veterinarians which may be needed more urgently elsewhere) and farmer welfare and behaviour (in response to the fear for potential loss of livestock and livelihood).

The type of premises where the incursion occurs can drastically impact on the scale of both of these biases and, therefore, on the resilience of predictions to temporal uncertainties. In the Scottish swine industry, the predictability of the number of premises infected via animal movement (Fig. 1c) and of specific premises that become infected (Fig. 1d) differ whether epidemics are generated by commercial or non-commercial producers. While our results indicate that all inferences produced from dynamic network models clearly suffer when the time of infection is estimated earlier (Figs 1d and 2), more conservative estimates of time of infection appear only preferable when incursion occurs in small producers. In this situation, widening the time window considered for the incursion would ensure that the incursion is included while not losing performance. Although this may be counterintuitive, it could be explained by the frequency of movements occurring from small producers. It has been previously shown that the rate of movement from and to small producers in Scotland is four to ten times lower than commercially-driven producers^20^, with an average of a movement every 29 weeks. It is therefore likely that increasing the time window for the incursion would include most of the movements that may be infectious while avoiding the inclusion of a large number of farms that are not infected. These results suggest that widening the time window considered for the incursion would provide a cost-efficient strategy when responding to incursion of infectious diseases in small producers, avoiding wasting resources that would be required to establish a precise incursion date.

In the model, we have first assumed that the trade of at least one animal between infected and susceptible premises was sufficient to allow infection to occur. It is obvious that this assumption may overestimate the extent of disease spread via movements of pigs (although bearing in mind that this model did not consider the potential for spread by other routes), as the infection process between farms is stochastic and depends on the within-farm prevalence as well as the virulence of the relevant outbreak strain. However, these assumptions seem appropriate because they not only increase the ease of the comparison between epidemic trees, but also enable (1) robust estimates of the potential geographical extent of disease spread that is consistent with contact tracing procedures and (2) communication of the general implication of temporal uncertainties in model inferences to policy makers (and model users in general). Nevertheless, varying the probability of transmission did not change the qualitative outcome of our analysis (Fig. 3).

It is clear from this study that on detection of an incursion, effort should be focused on obtaining an accurate incursion date. Improved accuracy of this estimate will improve the validity of epidemiological outputs from dynamic network models at early stages of an epidemic, and therefore will optimise the identification of the sources of infection and any presumed susceptible in-contact animals. However, quick detection of disease incursions is also critical. While the role of small producers in the spread of swine diseases has been previously shown^28^,^29^, routine surveillance activities (i.e. surveillance conducted not during an outbreak) mostly target assured commercial producers (for example abattoir inspection, veterinary/health scheme monitoring). Superficially, this risk-based surveillance strategy is reasonable because of the important influence of commercial producers on the sustainability of pig products (and the pig industry) and thus, food security^30^. However, Fig. 4 suggests that exclusively targeting assured commercial producers during routine surveillance activities will likely miss incursion events in backyard producers. Simulation studies looking at the spread of CSF in Bulgaria, where small producers are believed to play a role in the persistence of the disease^31^, have shown that infections from small producers to assured producers were rare^32^. Although consistent with our findings (Fig. 4), our results also indicated that non-assured commercial producers may constitute a bridge of infection between the non-commercial and commercial sectors of the swine industry in Scotland. With regards to improving surveillance for incursions of emerging swine diseases in Scotland, non-assured commercial producers may represent a sentinel population which would allow the detection of incursions in the non-commercial sector of the industry.

In this study, we assessed the usefulness of national electronic animal movement databases as a tool for traceability by examining the degree to which uncertainty around incursion time may affect predictions on the pre-detection spread of emerging swine diseases such as CSF and ASF in Scotland. Our results on movement patterns of swine in Scotland are also important for other exotic diseases of swine (e.g. foot-and-mouth disease) and may have relevance for other swine industries. Although the pig industry in Scotland is small, commercial production is well organised and focuses on assured production of high quality farrow-to-finish pigs. The pig industry in Scotland also shows a relative high diversity of producer types, with a large proportion of non-commercial pig holdings^20^. The Scottish swine industry may then represent a good example for similar industries, where non-commercial pig farming has an important place.

In Scotland, movements of swine shows a lack of seasonality^20^, similar to what has been reported in other countries^12^,^33^. It may therefore be possible to extrapolate these results to other similarly structured pig populations. In contrast, more work is required to determine whether these findings are applicable to other livestock sectors. The magnitude and directionality of movements of cattle and sheep in Scotland are highly seasonal. As such, these patterns will likely have an impact on the probability of epidemic take off^5^,^11^, and therefore are also likely to affect the predictability of the network structure in these sectors.

In conclusion, the type of premises and the uncertainty of the time of disease incursion will affect dynamic network model accuracy and thus, usefulness. Cursorily, it may appear that if the incursion time is uncertain, using conservative estimates of incursion time (i.e. covering a wider time window) would increase the probability of detecting all potentially infected farms. However, this approach also generates a larger number of premises that would require field-based investigation (of which a higher proportion would be negative), which would be challenging when resources are limited. Resources may be better placed trying to more accurately determine the incursion time, since dynamic network models can make valuable predictions to help with disease control and resource allocation if the incursion time is known. In such situation, efforts in improving surveillance prior to disease incursion are critical to optimise responses to disease incursions.

## Methods

### Data

All movement data were extracted from the Scottish livestock electronic identification and traceability database (ScotEID) which came into use in November 2011. We refer to^20^ for further details on the data collection, process and quality as well as some preliminary descriptive analyses.

Briefly, under Scottish (SSI 2011/351) and European legislation (Commission Decision 2000/678/EC), all pig keepers moving animals are required to register online with ScotEID and electronically record any movements ahead of time. To avoid selection bias due to inevitable missing or non-reported movements in the early stages of implementation of the database, we restricted our analysis to all movements recorded from January 1^st^ 2012 to May 31^st^ 2013. We used January 1st 2012 for the start of the study period, on the basis that (1) it corresponds to the time when the previous movement database (the Scottish Animal Movement System, SAMS) recording Scottish animal movements ended (i.e. November 2011), and (2) there has been a stabilisation of the movement pattern since December 2011.

The database provides a comprehensive picture of all movements of pigs in Scotland at the batch level. As such, each movement record reports the County Parish Holding (CPH) identifier and postcode for departures and destinations, the number of animals involved, and the date of the movements. Details of premises type for departures and destinations are recorded in the movement database, allowing slaughterhouses, markets, show-grounds and ferry collection centres to be differentiated from agricultural holdings. Note that all markets recorded in ScotEID operate as auctioneers holding dedicated sales/collections of pigs for onward consignment to a slaughterhouse, also named “red markets”. Collections of animals that are destined to be slaughtered are therefore regularly carried out in these markets, but remain separated from the other activities of such premises, particularly activities dedicated to sales of pigs between producers.

### Pig producer types

Through the CPH identifier, the ScotEID movement database was linked to the 2011 Scottish Agricultural Census, and to the 2010 GB Agricultural Census, to obtain information on the total number of pigs and sows present on farm. We further link the data to the 2013 Quality Meat Scotland (QMS) register (for Scottish premises) and to the 2013 Red Tractor register (for non-Scottish premises) to identify if producers were members of a health quality assurance scheme. Pig producers were then classified according to their pig population size, movement activity and the health quality assurance scheme membership^20^:

1. “Small pig producers”: agricultural holdings with an unknown number of pigs; or less than five sows, and/or less than 10 finishers; and showing no records of movements of more than 50 pigs within the study period.
2. “Non-assured commercial producers”: agricultural holdings with more than five sows and/or more than 10 finishers; or showing records of movements of more than 50 pigs during the study period, but do not belong to a quality health assurance scheme from QMS or Red Tractor, the main British assurance schemes.
3. “Assured commercial producers”: agricultural holdings with more than five sows and/or more than 10 finishers; or showing records of movements with more than 50 pigs during the study period but also belong to a quality health assurance scheme from QMS and/or Red Tractor.

### Infection path

The spread of disease within the Scottish swine industry was modelled using a simple stochastic discrete-time SI model. Our model treated each premises involved in the movement of Scottish pigs as a single unit. In this model, all premises are susceptible (S) to the infection at the start of the epidemics, except for a single premises, chosen at random, that would initially be at the infected, and infectious, state (I). During the course of an epidemic, disease passes from infected premises *i* to susceptible ones *j* via movements of pigs with a probability *M*_*ij,t*_ such as 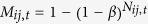, where *β* is the probability that a single pig from *i* may carry the disease and potentially transmit it to *j* (somewhat corresponding to the within-herd prevalence), *N*_*ij,t*_ the number of pigs that moved from *i* to *j* per time-step *t* and with movements of pigs synchronously updated at each time-step. Although other transmission routes have also been implicated in the spread of swine fevers (such as spread via fomites, wild boar, semen or pig products), only infection through live pig movements was considered as it the most common transmission route^17^,^18^. Here, the model is seeded at incursion time *t*_0_, progresses in discrete time steps *t* of one day, and runs for a fixed period *T*.

In addition to swine producers, gathering places (e.g. markets, show grounds, and ferry collection centres) were considered in the spread of diseases. Regulations are in place in Scotland, as in most EU countries, to ensure that the spread of pathogens via movements of animals through gathering places is limited. Gathering places should not keep pigs overnight and have cleaning and disinfection implemented after each day of activity (Council Directive 97/12/EC). As such, the model considers that all infected gathering places would go back to the susceptible state after one day (thereby following a SIS process), whereas infected swine producers would remain infected for the remaining of the simulation period *T*. As a consequence, epidemics were considered starting by a swine producer only. The model was used only to look at the spread of disease before detection. Therefore the control measures that would be initiated on identification of the disease (such as culling of pigs on infected premises, movement restrictions) were not included in the model.

To ensure that only the heterogeneity and the structure of the dynamic network formed by the movements of pigs were driving the modelled epidemics, all swine producers involved in the movements of pigs were considered identical, such that their producer type or herd size would not have any effect on the transmission dynamics. Unless otherwise stated, we considered the extreme case where infection occurs if at least one animal from an infected premises is received by a susceptible one, i.e. when *β* = 1. It is obvious that, given such a model structure, the characteristics of simulated epidemics would be overestimated and would not reflect the intrinsic potential of disease spread in the Scottish swine industry. However, such a model provides information on the maximum infection tree generated by each index-case via movements of animals, which not only provides an estimate of the maximum epidemic size generated by the movement of animals for the considered *T*, but also identifies all premises that are likely to be infected. Furthermore, such a model structure provides an estimate of infection trees for each incursion location that is easily comparable between time periods.

## Additional Information

**How to cite this article**: Porphyre, T. *et al.* Using national movement databases to help inform responses to swine disease outbreaks in Scotland: the impact of uncertainty around incursion time. *Sci. Rep.* **6**, 20258; doi: 10.1038/srep20258 (2016).
